# Mineralogical and geochemical characteristics of the Nkoteng-Mbandjock alluvial clays from Sanaga valley deposits (Cameroon, Central Africa): Implications for source weathering and provenance

**DOI:** 10.1016/j.heliyon.2024.e28395

**Published:** 2024-03-21

**Authors:** Elisé Sababa, Natanael Tehna, Beyanu Anehumbu Aye, Morine-Majolie Manfotang Chiozem, Armel Zacharie Ekoa Bessa, Ehbeudeu Kanewene, Njilah Isaac Konfor

**Affiliations:** a Department of Earth Sciences, University of Yaoundé I, P.O. Box 812, Yaoundé, Cameroon; b Department of Mining and Mineral Engineering, National Higher Polytechnic Institute (NAHPI), The University of Bamenda, P.O. Box 39, Bambili, Cameroon; c Department of Earth Sciences and Environment, Higher Teacher Training College, University of Bertoua, P.O. Box 652, Bertoua, Cameroon

**Keywords:** Shale, Source area-weathering, Immature sediment, Oxic environment, Felsic source, Sanaga valley

## Abstract

This research investigates the physico-chemical, mineralogical and geochemical attributes of alluvial clayey sediments in the Nkoteng-Mbandjock regions of the Sanaga valley, Cameroon. The primary objective is to elucidate the source area-weathering and provenance of these sediments. Grain size distribution analyses were conducted using the Robinson-Kӧln's pipetting method. The physico-chemical parameters were evaluated by an HACH-HQ11d brand electric pH meter, while the mineralogical compositions were determined by X-ray Diffraction. Major and trace element concentrations were measured employing X-ray Fluorescence and Inductively Coupled Plasmas-Mass Spectrometry. Textural classification identified the Sanaga valley alluvial clay deposits as predominantly silty clayey and clayey muddy. Geochemical classification diagram positioned them in the shale and Fe-shale fields. Weathering indices of alteration exhibited a consistent trend indicating a high degree of weathering in the source rock. A low Na_2_O/K_2_O ratio (average 0.18) and a high Index of Compositional Variability (ICV; average 2.29) suggested immature sediments. Additionally, low SiO_2_/Al_2_O_3_ ratio (average 3.93) implied an origin from stable settings with recycled inputs. These characteristics were further supported by elemental ratios such as Zr/Sc (average 47.12), U/Th (average 0.24) and Th/Sc (average 1.48). Furthermore, indicators like V/Cr (average 1.17), U/Th (average 0.24) and authigenic U (average −1.67) values suggested the deposition of the Sanaga valley alluvial clay under oxic conditions. The collective analysis of major and trace element distribution revealed felsic sources with minimal contributions from mafic rocks. These findings contribute to a comprehensive understanding of the geological processes and conditions influencing the composition and characteristics of the studied alluvial clay deposits in the Sanaga valley.

## Highlights

• We characterize alluvial clays from Sanaga valley deposits in Central Africa.
• These sediments result from complete weathering of felsic and mafic rock sources.
• They show high concentrations in Si, Al, Fe, Ba, Zr with negative Eu-anomalies.
• The deposition of the Sanaga valley alluvial clay occurred under oxic condition.

## 1 Introduction

The geochemical features of clastic sediments are influenced by various factors, including the composition of source material, (palaeo)weathering, (palaeo)climate, transportation, provenance, and tectonics [[1], [2], [3]]. Fine-grained fluvial sediments transported by large rivers such as Sanaga in Central Cameroon offer valuable chemical insights into terrestrial materials, weathering processes, provenance, sorting mechanisms, erosion patterns, and the depositional history of sediments [[4], [5], [6], [7], [8]]. Geochemical analyses of fine-grained sediments consistently reveal elevated concentrations of SiO_2_, Al_2_O_3_ and Fe_2_O_3_. Additionally, these sediments consist of secondary and accessory primary minerals capable of hosting significant quantities of immobile elements, including Ti, Al, Th, Zr, and Rare Earth Elements (REEs). Notably, these elements are predominantly present in the suspended load of the river and subsequently deposited as fine-grained overbank sediments [1, 9].

Clay materials, derived from geological sources, represent a commonly exploited resource for human well-being. Morphological, mineralogical and geochemical characterizations of these materials provide valuable insights into sedimentological processes and offer crucial information for their industrial applications. The grain size distribution and major element geochemistry of clay materials contribute to a robust classification of fine sediments [10,11]. The distribution patterns of major and trace elements serve as valuable indicators for determining weathering conditions and discerning the origin of source materials [12,13].

The Sanaga River, boasting a length of 918 km, stands as the longest river in Cameroon. The Sanaga basin harbors source rocks of diverse nature and expose sediments to varying climatic and weathering influences. This study delves into specific characteristics of alluvial clays in the Nkoteng-Mbandjock areas, aiming to facilitate a reasoned discourse on the deposition and provenance of the Sanaga valley alluvial clay deposits.

## 2 Geography and geology

The Nkoteng-Mbandjock areas are situated in the expansive peneplain that extends across South and Central Cameroon in Central Africa. Specifically, Mbandjock is positioned between 4°21′0'' and 4°34′30'' North latitude and 11°51′0'' and 12°0′0'' East longitude, while Nkoteng is located between 04°30 ′00'' and 04°36′00'' North latitude and 11°56′28'' and 12°03′90'' East longitude. The prevailing climate in this region is of the equatorial transitional type, characterized by two dry and two rainy seasons of unequal duration [14]. The average annual rainfall is recorded at 1495 mm, with a mean temperature of approximately 25 °C. Relative humidity ranges from 70 to 80%, varying across different locations. The dominant vegetation comprises savannah intersected by gallery forests, incorporating residual islands and semi-deciduous forest. The Sanaga basin, where the study area is located, falls in the "African Surface I" which spans almost the entire Central and South Cameroon [15]. This surface exhibits a gently undulating peneplain with altitudes ranging from 600 to 1000 m. The Sanaga River, the longest river in Cameroon at 918 km, irrigates an area of approximately 140,000 km^2^. Originating from the Adamawa plateau, it flows southwestward before discharging into the Atlantic Ocean. The environmental conditions in the region favor the development of ferralitic and hydromorphic soils in the flood plains.

**Fig. 1:**
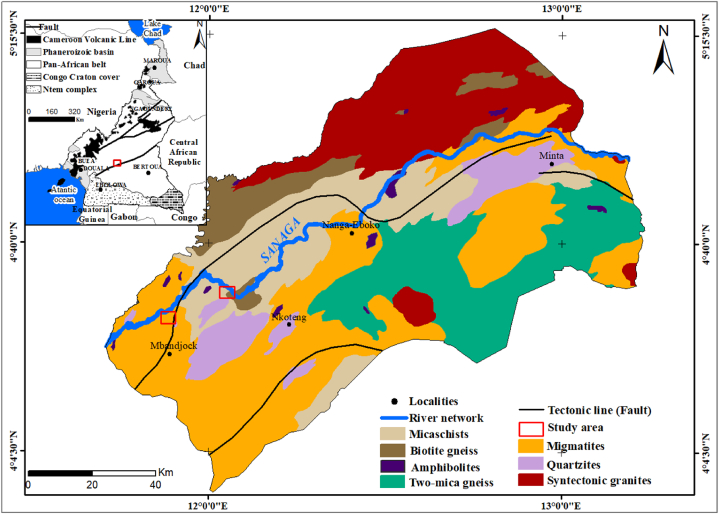
Geological map and location of the study area with the inset showing the general map of Cameroon.

The Sanaga River flows along a SSW-NNE fault which occurred in the Proterozoic during the Pan-African orogeny [56]. It flows predominantly over metasediments (2.1 Ga), Paleoproterozoic amphibolites and orthogneisses, granitoids from the Adamawa-Yade batholith (AYB), sedimentary clastic rocks of Cretaceous age, and Cenozoic rocks of the Cameroon Volcanic Line [16]. Meta-igneous unit predominantly comprises garnet gneisses, micaschists and pyroxenites. This unit occurs with the meta-sedimentary unit, which is characterized by variable gneissose rocks [17]. Low grade Panafrican metamorphic rocks (phyllites, shales, and siliceous facies represented by quartzites generally interbedded in shales) of the Lom series at the northeastern extremity of the Sanaga River basin and the medium to high-grade gneissic rocks of the Yaounde group [16,56]. Granitic gneisses are typical of the Panafrican basement of West Cameroon and Adamawa parts of the Sanaga river basin. The study area comprises diverse lithologies, including gneisses, quartzites, micashists, migmatites, granites, and amphibolites (Fig. 1).

## 3 Methodology

### 3.1 Sampling

A total of sixteen bulk clay samples, each weighing approximately 2 kg, were systematically collected from vertical sections of the Sanaga valley alluvial clay deposits in manually excavated pits. The samples were designated using a two-digit nomenclature: the first digit denotes the pit number, and the second digit signifies the layer, with "1" representing the upper layer and "2" indicating the bottom layer. The dimensions and characteristics of each layer are detailed in Table 1. Each excavation pit covered a surface area of 1 m × 1 m, with the depth varying based on the thickness of the clay deposits.

**Table 1:** Color, particle size distribution, texture, pH, Eh and EC of the Nkoteng-Mbandjock alluvial clays.

Pits	Mbandjock site									Nkoteng site								
	Profile ME 1		Profile ME 4		Profile ME 5		Profile ME 6		Av.	Profile NM 1		Profile NM 3		Profile NM 5		Profile NM 7		Av.
Samples	ME 11	ME 12	ME 41	ME 42	ME 51	ME 52	ME 61	ME 62	–	NM 11	NM 12	NM 31	NM 32	NM 51	NM 52	NM 71	NM 72	–
Depth (cm)	5–90	90–200	7–55	55–195	5–150	150–205	5–30	30–140	–	10–120	120–250	5–100	100–150	5–100	100–150	5–40	40–70	–
Munsell color	White (N8/)	Light red (2.5YR7/6)	Yellowish red (5YR5/6)	Reddish brown (2.5YR5/6)	Light gray (10YR7/2)	Light reddish gray (2.5YR7/1)	White (N8/)	Light bluish gray (5PB8/1)	–	Brownish yellow (10YR6/6)	Gray (10Y5/1)	Pale yellow (2.5Y8/3)	Light gray (2.5YR7/1)	Very dark gray (2.5YR3/1)	Dark brown (7.5YR3/3)	Light gray (2.5Y7/1)	Pale yellow (2.5Y7/3)	–
Clay (%)	60.60	65.72	39.28	33.02	48.21	71.17	44.14	54.29	52.05	32.00	56.96	41.77	36.97	40.71	39.04	50.65	42.24	42.54
Silt (%)	12.49	25.19	33.98	35.53	31.03	16.62	12.29	17.80	23.12	24.91	25.12	27.85	30.43	52.28	41.66	28.88	19.79	31.37
Sand (%)	26.89	9.07	26.74	31.45	20.74	12.19	43.56	27.90	24.82	42.56	17.91	5.80	32.57	7.00	19.30	20.14	37.96	22.91
pH	4.24	4.34	5.77	5.10	5.02	4.60	4.57	4.44	4.76	5.33	5.45	4.84	5.17	5.55	5.42	5.27	5.14	5.27
Eh (mV)	111.80	81.10	36.30	−1.00	29.80	12.60	42.40	70.20	44.90	44.70	24.60	21.40	37.90	−4.30	93.10	99.30	11.50	41.03
EC (dS/cm)	413.00	315.00	100.70	85.10	141.30	245.00	144.30	147.50	198.99	93.90	77.90	108.30	253.00	182.60	129.00	98.80	27.80	121.41

### 3.2 Analytical procedures

The colors attributed to the diverse clay samples, as outlined in Table 1, were determined using the Munsell color chart. Grain size distribution analyses were conducted utilizing the Robinson-Kӧln's pipetting method [18]. The physico-chemical attributes of the clay samples were assessed following the methodology elucidated by Sababa et al. [19] at the University of Yaoundé I (Cameroon). Specifically, 10 g of each sample were weighed and introduced in 100 mL beakers, which were subsequently filled with distilled water and homogenized for a period ranging from 15 to 30 min. The pH (0–14) and redox potential (Eh) were then measured using an electric pH meter from HACH-HQ11d. Electrical conductivity (EC) was determined using a conductivity meter from HACH brand.

Mineralogical and geochemical analyses were done at the Geoscience Laboratories of Sudbury (Canada) after sample preparation at the Department of Earth Sciences (University of Yaoundé I, Cameroon). The analytical methodologies for determining mineralogical and geochemical composition were previously documented in previous studies [19,20]. Mineralogical compositions were elucidated through X-ray Diffraction (XRD) using a PAN Analytical X’PERT PRO diffractometer coupled with a monochromator. The quantitative assessment of minerals in the various samples employed the Profex 4.3.6 software, which utilizes the Rietveld method. This method employs a least squares approach to refine a theoretical line profile until it matches the measured profile, accounting for instrumental broadening, wavelength dispersion, specimen function, and background function. Standards, with error margins between 5 and 10%, were used.

To determine the loss on ignition (LOI), samples underwent heating to 105 °C for water removal and 1000 °C to eliminate volatiles and oxidize iron. Following ignition, major element contents were assessed using a Rigaku RIX-3000 wavelength-dispersive X-ray fluorescence spectrometer. Concentrations of trace elements were determined through Inductively Coupled Plasma-Mass Spectrometry (ICP-MS) following acid digestion in closed beakers. The PerkinElmer 5000 ICP-MS spectrometer was employed for this purpose, with instrument precision ranging between 5 and 8.5% based on element contents.

### 3.3 Calculation of some alteration indices

Chemical Index of Alteration (CIA = [Al_2_O_3_/(Al_2_O_3_ + Na_2_O + CaO* + K_2_O)] × 100) is calculated with the molecular contents of oxides. It quantifies weathering processes of source rocks [21]. CaO* is the CaO content of silicate minerals in sample which is the lower value between Na_2_O and [CaO-(10/3)P_2_O_5_] molar contents [22]. The CIA is considered as the ratio of the most mobile elements to the least mobile elements. Low CIA is associated with low degree of weathering. Fresh rocks should have a CIA value of about 50% or less. The removal of all mobile cations (K^+^, Na^+^ and Ca^2+^) compared to relatively stable Al^3+^ in supergene environments is associated with a value of 100% [22].

Plagioclase index of alteration (PIA) is also used to assess the weathering degree of the source rocks [23]. It is calculated (PIA = [(Al_2_O_3_ - K_2_O)/(Al_2_O_3_ + CaO* + Na_2_O - K_2_O)] × 100; [24]) with oxides molar contents and use CaO* like CIA calculation. According to Bhaskar et al. [23], the minimum value of PIA is about 50%, as a result of low plagioclase weathering. Its highest value is 100% which indicates complete hydrolysis of plagioclase.

### 3.4 Assessment of rare earth element fractionation

The total rare earth elements (REE: La, Ce, Pr, Nd, Sm, Eu, Gd, Tb, Dy, Ho, Er, Tm, Yb and Lu), light rare earth elements (LREE: La, Ce, Pr, Nd, Sm and Eu) and heavy rare earth elements (HREE: Gd, Tb, Dy, Ho, Er, Tm, Yb and Lu) were systematically calculated. In order to provide a comprehensive understanding of the rare earth elements' behavior in the investigated materials, calculations pertaining to specific fractionation degrees [[25], [26], [27]] were conducted. Notably, the data for chondrite were referenced from Pourmand et al. [28].

The following equations have been used:

(1) Ce anomaly: Ce/Ce*= (Ce_sample_/Ce_chondrite_)/(La_sample_/La_chondrite_)^1/2^(Pr_sample_/Pr_chondrite_)^1/2^;
(2) Eu anomaly: Eu/Eu*= (Eu_sample_/Eu_chondrite_)/(Sm_sample_/Sm_chondrite_)^1/2^(Gd_sample_/Gd_chondrite_)^1/2^;
(3) REE fractionation: (La/Yb)_N_= (La_sample_/La_chondrite_)/(Yb_sample_/Yb_chondrite_);
(4) LREE fractionation: (La/Sm)_N_=(La_sample_/La_chondrite_)/(Sm_sample_/Sm_chondrite_);
(5) HREE fractionation: (Gd/Yb)_N_=(Gd_sample_/Gd_chondrite_)/(Yb_sample_/Yb_chondrite_)

## 4 Results

### 4.1 Morphology, physico-chemistry and mineralogy

**Fig. 2:**
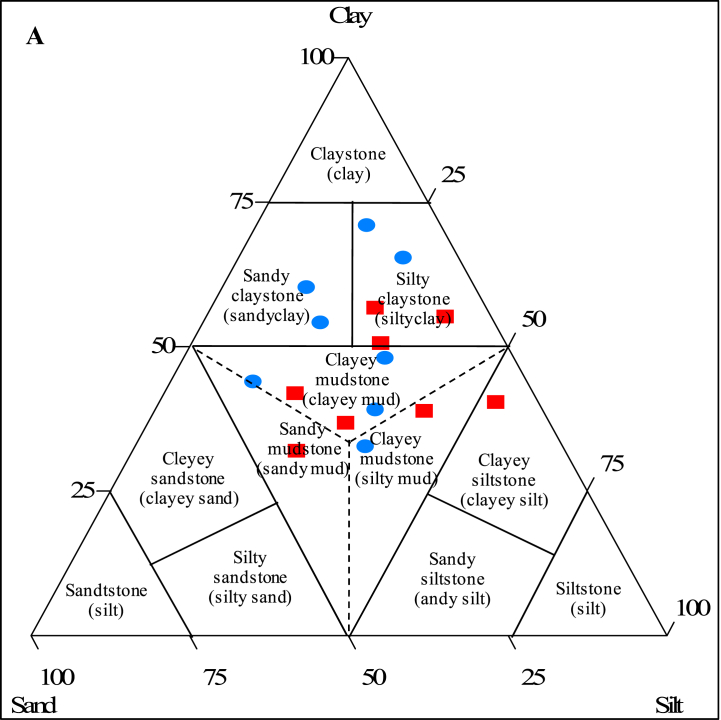
Plots of the Nkoteng-Mbandjock alluvial clays in the clay-sand-silt diagram for characterization of texture. Circles represent samples from Mbandjock and squares represent samples from Nkoteng.

Table 1 provides a concise summary of the detailed morphology and physico-chemistry of the Sanaga valley alluvial clay deposits. The primary distinguishing factor between the two layers in each pit is their color, exhibiting both vertical and horizontal variations. Pit depths range from 70 to 250 cm (see Table 1). Beneath the Sanaga valley alluvial clay deposits lies a layer of silty sand enriched in organic matter, with a lower stratum composed of whitish brown sand. Grain size distribution analysis reveals a variable composition, with approximately 50% clay (<2 μm). Notably, samples from Mbandjock exhibit a higher clay content compared to those from Nkoteng, which are proportionally richer in silt (2–60 μm) (Table 1). According to Picard's (1971) textural classification, the Sanaga valley alluvial clay deposits primarily fall into the categories of silty clayey and clayey muddy (Fig. 2).

**Fig. 3:**
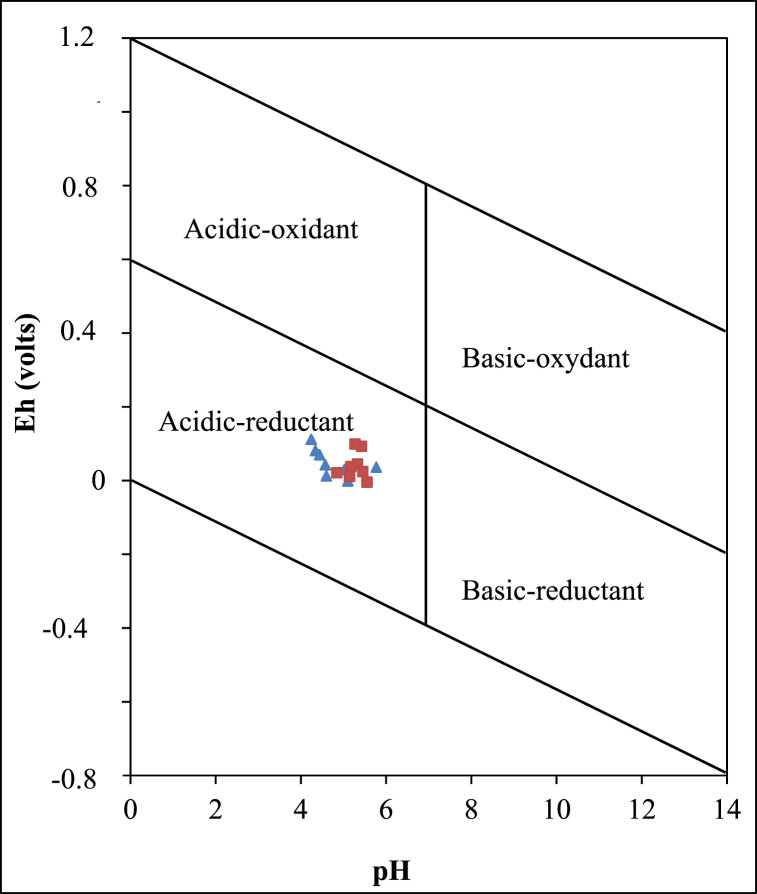
Eh-pH diagram [49] of the Nkoteng-Mbandjock alluvial clays. Triangles represent samples from Mbandjock and squares represent samples from Nkoteng.

The Sanaga valley alluvial clay deposits exhibit a moderate acidity, with samples from Mbandjock (average pH 4.76) displaying greater acidity than those from Nkoteng (average pH 5.27). Additionally, the samples present variable values of redox potential (Eh) and Electrical Conductivity (EC). The pH *vs.* Eh plot indicates that the current conditions of the Sanaga valley alluvial clay deposits are characterized by acidity and reducing conditions (Fig. 3). Based on EC values, the alluvial clay deposits in the Sanaga valley are classified as strongly saline materials.

**Fig. 4:**
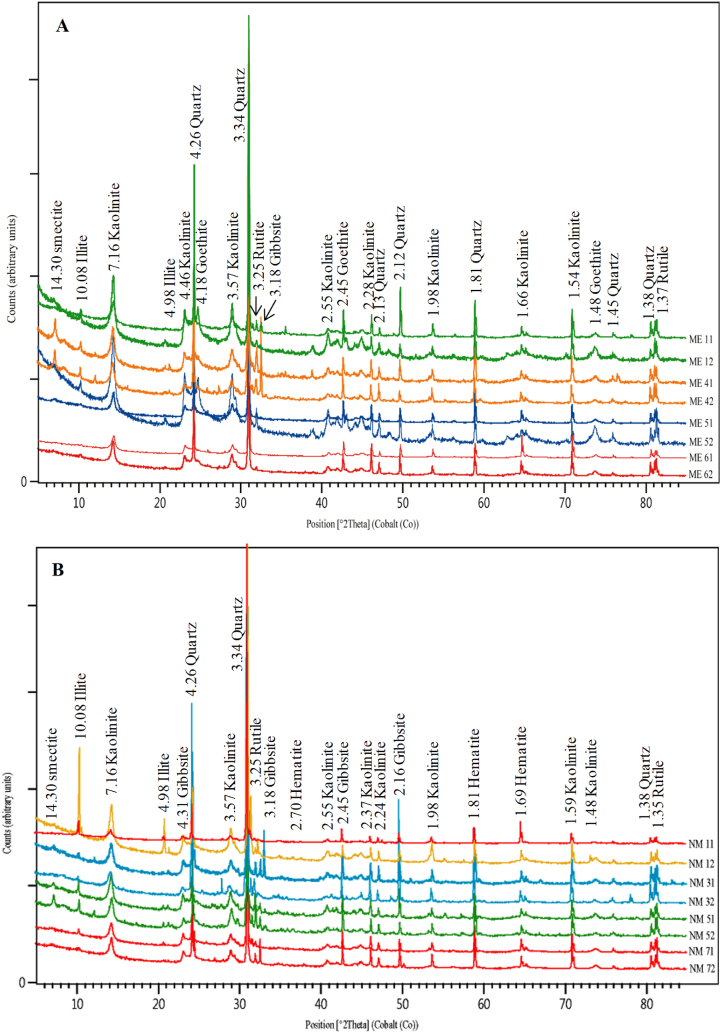
X-ray diffraction patterns of the Nkoteng-Mbandjock alluvial clays: A) Samples from Mbadjock; B) Samples from Nkoteng.

The mineralogical composition is predominantly comprised of kaolinite, quartz, illite, gibbsite, rutile and goethite/hematite (Fig. 4). Kaolinite (47–71%), quartz (12–41%), illite (6-13), and rutile (1–5%) exhibit similar contents in both study sites (Table 2). Goethite (Fig. 4A) is notably more abundant in the Mbandjock site (average 3%), while hematite (Fig. 4B) is associated with samples from Nkoteng (average 1%) (Table 2). Smectite is present in trace amounts in select samples from both sites (Fig. 4; Table 2). The observed variation in color may be linked to the diverse composition of clay minerals (kaolinite and illite), as well as the presence of iron oxides and hydroxides.

**Table 2:** Mineralogical composition (in %) of the Nkoteng-Mbandjock alluvial clays.

	Mbandjock site									Nkoteng site									Statistical data		
	ME11	ME12	ME41	ME42	ME51	ME52	ME61	ME62	Av.	NM11	NM12	NM31	NM32	NM51	NM52	NM71	NM72	Av.	Min.	Max.	Av.
Quartz	41	18	30	35	22	12	22	21	25	28	13	26	28	18	30	22	25	24	12	41	24
Illite	6	6	6	8	8	8	7	7	7	11	13	7	7	10	7	8	8	9	6	13	8
Kaolinite	49	66	52	45	64	71	63	63	60	54	65	58	56	60	51	61	59	59	47	71	59
Smectite	–	–	3	4	–	–	–	–	4	–	–	1	–	3	2	–	–	2	1	4	3
Hematite	–	–	–	–	–	–	–	–	–	–	1	–	–	–	1	1	1	1	1	1	1
Goethite	2	7	4	3	–	4	1	1	3	–	–	–	–	–	–	–	–	–	1	7	3
Gibbsite	1	1	1	3	6	4	6	6	4	7	6	7	7	7	7	6	6	7	1	7	5
Rutile	1	2	4	2	–	1	1	2	2	1	2	1	1	2	2	2	1	2	1	5	2
Total	100	100	100	100	100	100	100	100	–	100	100	100	100	100	100	100	100	–	–	–	–

### 4.2 Geochemistry

#### 4.2.1 Major elements

The major elements in the alluvial clays from the Sanaga valley have wide range concentrations, as delineated in Table 3. Silica (SiO_2_) content spans a wide range (50–77 wt%), with an average value of 65 wt%. Alumina (Al_2_O_3_) follows as the second most abundant element, ranging from 11 to 25 wt% and averaging 16 wt%. The concentration of iron oxide (Fe_2_O_3_) varies between 1 and 10 wt%, with samples from the Mbandjock site displaying the highest average Al_2_O_3_ and Fe_2_O_3_ contents (19 wt% and 5 wt%, respectively). TiO_2_ concentrations range from 1 to 3 wt%. Most samples from both Mbandjock and Nkoteng sites exhibit K_2_O values exceeding 1 wt%, while the contents of other oxides (MgO, CaO, Na_2_O, and MnO) are predominantly below 1 wt% (Table 3). Loss on ignition (LOI) varies from 3 to 12 wt% with an average content of 8 wt%. The lowest LOI average value is recorded in the sample from the Nkoteng site. The mean values of SiO_2_/Al_2_O_3_ (3.93), Al_2_O_3_/TiO_2_ (10.05), and notably Al_2_O_3_/Na_2_O (111.40) ratios are elevated, whereas Na_2_O/K_2_O ratios are low, averaging 0.18 (Table 3).

**Table 3:** Distribution of major elements (wt.%) in the Nkoteng-Mbandjock alluvial clays.

		Mbandjock site									Nkoteng site									Statistical data		
	dl	ME11	ME12	ME41	ME42	ME51	ME52	ME61	ME62	Av.	NM11	NM12	NM31	NM32	NM51	NM52	NM71	NM72	Av.	Min.	Max.	Av.
SiO_2_	0.04	67.43	50.90	57.58	64.80	67.42	51.69	71.59	67.39	62.35	74.31	61.25	65.63	77.18	57.16	64.41	66.81	75.88	67.83	50.90	77.18	64.93
Al_2_O_3_	0.02	17.03	23.40	18.89	15.64	16.64	24.85	16.50	16.95	18.74	13.57	21.77	16.55	11.51	20.48	15.68	17.30	12.80	16.21	11.51	24.85	17.55
Fe_2_O_3_(t)	0.01	3.45	9.51	7.56	5.94	2.69	7.46	1.73	4.26	5.33	2.42	2.07	4.48	1.86	6.20	6.21	2.22	1.84	3.41	1.73	9.51	4.43
MgO	0.01	0.18	0.27	0.59	0.57	0.22	0.28	0.16	0.17	0.31	0.24	0.32	0.42	0.26	0.56	0.55	0.27	0.18	0.35	0.16	0.59	0.33
K_2_O	0.01	1.48	0.91	1.94	2.39	0.81	0.78	0.37	0.46	1.14	1.36	1.34	2.10	2.24	2.01	2.38	1.22	1.01	1.71	0.37	2.39	1.41
Na_2_O	0.02	0.27	0.10	0.44	0.60	0.11	0.10	0.07	0.07	0.22	0.27	0.26	0.38	0.49	0.39	0.60	0.14	0.14	0.33	0.07	0.60	0.27
CaO	0.01	0.12	0.05	0.36	0.48	0.11	0.11	0.03	0.03	0.16	0.07	0.09	0.26	0.29	0.32	0.48	0.10	0.08	0.21	0.03	0.48	0.18
TiO_2_	0.01	1.45	1.91	1.81	1.72	2.70	1.63	1.58	2.05	1.86	1.93	1.81	1.58	1.10	1.70	1.62	2.22	1.75	1.71	1.10	2.70	1.79
MnO	0.00	0.01	0.01	0.07	0.06	0.02	0.01	0.01	0.02	0.03	0.02	0.02	0.03	0.03	0.04	0.04	0.02	0.02	0.02	0.01	0.07	0.03
P_2_O_5_	–	0.03	0.07	0.14	0.12	0.07	0.08	0.04	0.06	0.08	0.05	0.06	0.09	0.06	0.20	0.17	0.10	0.06	0.10	0.03	0.20	0.09
LOI	–	7.24	11.89	9.24	6.58	7.97	11.94	7.90	8.44	8.90	5.57	9.66	7.42	3.99	10.19	7.77	8.17	5.64	7.30	3.99	11.94	8.15
Total	–	98.69	99.02	98.62	98.9	98.76	98.93	99.98	99.9	99.12	99.81	98.65	98.94	99.01	99.25	99.91	98.57	99.4	99.18	–	–	–
SiO_2_/Al_2_O_3_	–	3.96	2.18	3.05	4.14	4.05	2.08	4.34	3.98	3.47	5.48	2.81	3.97	6.71	2.79	4.11	3.86	5.93	4.46	2.08	6.71	3.93
Na_2_O/K_2_O	–	0.18	0.11	0.23	0.25	0.14	0.13	0.19	0.15	0.17	0.20	0.19	0.18	0.22	0.19	0.25	0.11	0.14	0.19	0.11	0.25	0.18
Al_2_O3/TiO_2_	–	11.74	12.25	10.44	9.09	6.16	15.25	10.44	8.27	10.46	7.03	12.03	10.47	10.46	12.05	9.68	7.79	7.31	9.60	6.16	15.25	10.05
Al_2_O_3_/Na_2_O	–	63.07	234.00	42.93	26.07	151.27	248.50	235.71	242.14	155.46	50.26	83.73	43.55	23.49	52.51	26.13	123.57	91.43	61.83	23.49	248.50	111.40
ICV	–	2.45	1.83	1.48	1.33	2.50	2.40	4.18	2.40	2.32	2.15	3.69	1.79	1.84	1.83	1.32	2.80	2.55	2.25	1.32	4.18	2.29
CIA (%)	–	88.30	95.00	84.40	77.90	93.10	95.50	96.70	96.10	90.80	86.90	91.40	83.10	75.40	85.80	77.90	90.90	89.70	85.14	75.40	96.70	88.18
PIA (%)	–	95.90	98.90	92.40	87.50	97.80	98.60	99.00	99.00	96.14	95.50	97.20	92.9	87.20	93.70	87.60	97.50	96.90	93.50	87.20	99.00	94.93

d.l.: detection limit.

LOI: loss on ignition.

CIA = [Al_2_O_3_/(Al_2_O_3_ + CaO∗ + Na_2_O + K_2_O)]*100 from Nesbitt and Young (1982).

ICV=(Fe_2_O_3_ + K_2_O + Na_2_O + CaO + MgO + TiO_2_)/Al_2_O_3_ from Cox et al. (1995).

PIA (%) = [(Al_2_O_3_ - K_2_O)/(Al_2_O_3_ + CaO + Na_2_O - K_2_O)] × 100 from Fedo et al. (1995).

MIA = 100 x [(Al_2_O_3_ + Fe_2_O_3_)/(Al_2_O_3_ + Fe_2_O_3_ + MgO + CaO* + Na_2_O + K_2_O)] from Babechuk et al. (2014).

#### 4.2.2 Trace elements

The concentration of trace elements in the alluvial clays from the Sanaga valley is shown in Table 4. Barium exhibits the highest content, ranging from 175 to 944 ppm, with a mean value of 597 ppm. Zirconium follows as the second most abundant element, varying between 304 and 775 ppm, with an average content of 556 ppm. Mean values for V (116 ppm), Sr (101 ppm), and Cr (100 ppm) are lower than those observed for the aforementioned trace elements. The samples from the Nkoteng site display the highest mean values for Ba, Zr, and Sr, while the Mbandjock site registers the highest average contents for V and Cr (Table 4). Considerable variations are also noted for Zn (34–88 ppm), Rb (17–63 ppm), Nb (57–723 ppm), and Ni (19–47 ppm) (Table 4). Notably, the Cr/V, Y/Ni, Rb/Sr, and U/Th values are exceptionally low (Table 4). Ratios such as Ni/Co, Zr/Sc, and to a lesser extent Th/Sc suggest that Zr, Ni, and Th are likely less mobile compared to Sc and Co in these environments (Table 4).

**Table 4:** Distribution of trace elements (ppm) in the Nkoteng-Mbandjock alluvial clays.

		Mbandjock site									Nkoteng site									Statistical data		
	d.l.	ME 11	ME 12	ME 41	ME 42	ME 51	ME 52	ME 61	ME 62	Av.	NM 11	NM 12	NM 31	NM 32	NM 51	NM 52	NM 71	NM 72	Av.	Min.	Max.	Av.
Ba	1.30	707.00	483.00	768.20	922.70	385.70	423.00	186.10	175.30	506.38	541.60	463.40	814.10	901.00	840.60	943.70	575.40	505.00	698.10	175.30	943.70	596.60
Sr	1.30	102.60	67.90	139.40	179.70	78.90	57.30	27.20	26.20	84.90	78.50	54.40	146.50	172.70	137.30	183.90	92.20	85.20	118.84	26.20	183.90	100.87
Li	0.24	22.20	26.50	20.10	15.00	21.10	32.90	22.90	22.90	22.95	30.90	54.70	19.80	17.10	25.60	16.90	28.50	26.40	27.49	15.00	54.70	25.09
Rb	0.15	35.22	26.58	41.96	46.47	30.04	17.47	24.24	25.41	30.92	58.75	26.29	46.37	59.72	63.26	55.67	55.28	50.22	51.95	17.47	63.26	40.82
Th	0.01	15.13	17.61	18.79	16.12	18.22	16.69	15.88	19.59	17.25	20.40	20.27	15.44	18.98	19.68	18.34	19.67	14.35	18.39	14.35	20.40	17.79
Zr	4.00	473.00	330.00	562.00	775.00	575.00	304.00	525.00	620.00	520.50	645.00	418.00	631.00	658.00	666.00	729.00	497.00	518.00	595.25	304.00	775.00	555.68
Nb	0.05	30.47	41.08	39.62	36.73	57.11	34.11	37.62	42.65	39.92	41.84	40.07	33.26	23.20	37.76	32.34	46.74	42.56	37.22	23.20	57.11	38.65
U	0.01	3.49	4.11	4.72	4.41	4.83	4.13	2.73	3.46	3.98	4.51	4.29	4.63	4.06	5.50	4.43	5.44	3.69	4.57	2.73	5.50	4.26
Hf	0.09	12.28	9.05	15.08	18.93	13.88	8.01	12.32	14.92	13.06	16.53	10.63	15.84	16.57	15.59	17.08	12.42	11.77	14.55	8.01	18.93	13.76
Cr	2.90	84.00	115.00	107.00	103.00	102.00	117.00	90.00	109.00	103.38	79.00	110.00	89.00	58.00	110.00	89.00	107.00	93.00	91.88	58.00	117.00	97.96
V	0.40	99.70	163.10	127.00	113.70	131.40	161.20	75.90	110.20	122.78	99.40	125.80	108.30	59.80	154.00	110.70	106.50	93.60	107.26	59.80	163.10	115.48
Ni	0.60	31.60	36.30	40.10	32.90	26.50	46.80	34.20	35.80	35.53	19.40	38.30	29.60	19.50	43.70	30.50	33.20	26.60	30.10	19.40	46.80	32.97
Zn	2.00	36.10	47.80	68.80	62.50	37.90	53.20	34.10	38.50	47.36	61.40	88.30	52.10	38.00	88.30	66.30	49.50	40.50	60.55	34.10	88.30	53.57
Cu	0.40	12.60	17.10	19.50	17.50	13.50	22.80	16.60	20.20	17.48	13.60	20.30	16.80	11.10	29.70	18.40	16.70	13.80	17.55	11.10	29.70	17.51
Co	0.09	8.79	10.18	17.32	15.99	7.03	13.20	7.34	7.71	10.95	4.80	9.20	7.78	5.14	14.61	13.57	9.08	7.40	8.95	4.80	17.32	10.01
Sc	0.17	11.20	13.40	12.10	10.40	12.70	14.30	13.00	14.20	12.66	9.20	13.90	10.60	8.40	17.70	11.20	14.20	11.60	12.10	8.40	17.70	12.40
Y	0.09	14.35	14.92	22.79	26.64	25.37	13.03	25.66	29.99	21.59	19.74	31.39	25.26	32.03	41.62	31.41	36.96	28.77	30.90	13.03	41.62	25.97
Ga	0.04	22.00	31.43	25.83	21.34	23.39	33.46	22.24	24.22	25.49	19.39	31.25	22.73	15.54	31.02	21.02	24.32	19.19	23.06	15.54	33.46	24.34
Pb	0.29	23.75	37.84	31.75	27.00	35.98	30.59	22.62	24.08	29.20	22.66	30.15	30.48	22.58	35.39	27.32	36.75	28.46	29.22	22.58	37.84	29.21
Zr/Sc	–	42.23	24.63	46.45	74.52	45.28	21.26	40.38	43.66	42.30	70.11	30.07	59.53	78.33	37.63	65.09	35.00	44.66	52.55	21.26	78.33	47.12
Th/Sc	–	1.35	1.31	1.55	1.55	1.43	1.17	1.22	1.38	1.37	2.22	1.46	1.46	2.26	1.11	1.64	1.38	1.24	1.60	1.11	2.26	1.48
Cr/V	–	0.84	0.71	0.84	0.91	0.78	0.73	1.19	0.99	0.87	0.79	0.87	0.82	0.97	0.71	0.80	1.00	0.99	0.87	0.71	1.19	0.87
Y/Ni	–	0.45	0.41	0.57	0.81	0.96	0.28	0.75	0.84	0.63	1.02	0.82	0.85	1.64	0.95	1.03	1.11	1.08	1.06	0.28	1.64	0.84
Rb/Sr	–	0.34	0.39	0.30	0.26	0.38	0.30	0.89	0.97	0.48	0.75	0.48	0.32	0.35	0.46	0.30	0.60	0.59	0.48	0.26	0.97	0.48
U/Th	–	0.23	0.23	0.25	0.27	0.26	0.25	0.17	0.18	0.23	0.22	0.21	0.30	0.21	0.28	0.24	0.28	0.26	0.25	0.17	0.30	0.24
V/Cr	–	1.19	1.42	1.19	1.10	1.29	1.38	0.84	1.01	1.18	1.26	1.14	1.22	1.03	1.40	1.24	1.00	1.01	1.16	0.84	1.42	1.17
Ni/Co	–	3.59	3.57	2.32	2.06	3.77	3.55	4.66	4.64	3.52	4.04	4.16	3.80	3.79	2.99	2.25	3.66	3.59	3.54	2.06	4.66	3.53
AU	–	−1.55	−1.76	−1.55	−0.96	−1.25	−1.43	−2.57	−3.07	−1.77	−2.29	−2.47	−0.52	−2.26	−1.06	−1.68	−1.11	−1.09	−1.56	−3.07	−0.52	−1.67

d.l.: detection limits.AU = X_U_-(Y_Th_)/3. X_U_ and Y_Th_ represent the contents of U and Th respectively (Wignall and Myers 1988).

#### 4.2.3 Rare earth elements

**Fig. 5:**
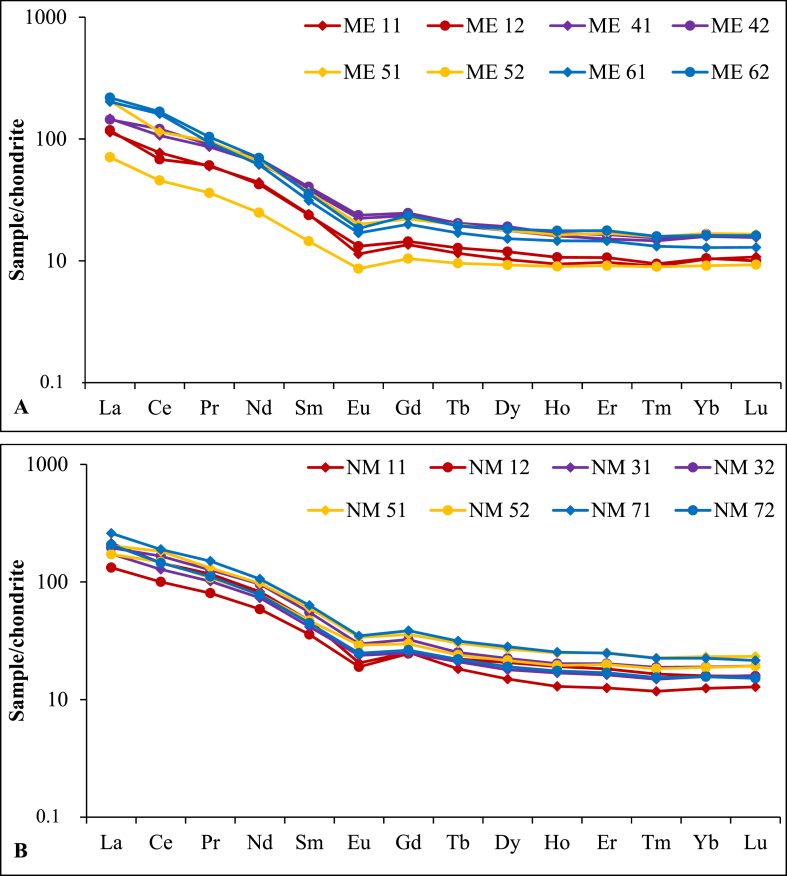
Chondrite-normalized REE patterns [28] for the Nkoteng-Mbandjock alluvial clays: A) Samples from Mbandjock and B) Samples from Nkoteng.

The concentrations of rare earth elements (REE) in the Sanaga valley alluvial clay deposits exhibit a range from 73 to 288 ppm with a mean value of 191 ppm (Table 5). The Nkoteng site displays slightly higher REE contents than those observed at the Mbandjock site. The alluvial clay deposits in the Sanaga valley are notably characterized by Light Rare Earth Element (LREE) enrichment, with an average of 173 ppm, in comparison to Heavy Rare Earth Element (HREE) with an average of 17 ppm across both sites. The LREE/HREE values span from 7 to 13, with a mean value of 10. Normalized patterns relative to chondrite [28] unveil negative anomalies in Eu for most samples from both sites (Fig. 5A and B), with an average Eu/Eu* ratio of 0.63 (Table 5). Slight negative and positive anomalies are also evident in Ce (Ce/Ce* ranging from 0.8 to 1.17) (Table 5). Ratios such as (La/Yb)_N_, (La/Sm)_N_, and (Gd/Yb)_N_ (Table 5) indicate low fractionation, especially for HREE as confirmed by the REE patterns (Fig. 5).

**Table 5:** Distribution of rare earth elements (ppm) in the Nkoteng-Mbandjock alluvial clays.

		Mbandjock site									Nkoteng site									Statistical data		
	dl	ME 11	ME 12	ME 41	ME 42	ME 51	ME 52	ME 61	ME 62	Av.	NM 11	NM 12	NM 31	NM 32	NM 51	NM 52	NM 71	NM 72	Av.	Min.	Max.	Av.
La	0.09	28.00	29.10	36.30	35.60	50.70	17.50	49.90	53.90	37.63	52.70	32.70	42.80	48.20	50.20	42.40	64.00	51.20	48.03	17.50	64.00	42.52
Ce	0.17	48.82	43.02	67.48	76.47	72.39	28.86	101.63	105.97	68.08	92.13	63.32	80.79	104.85	115.32	93.66	119.53	91.64	95.16	28.86	119.53	80.82
Pr	0.02	5.73	5.83	8.27	8.62	9.18	3.46	8.98	9.98	7.51	11.20	7.70	9.73	12.13	12.60	10.40	14.43	10.67	11.11	3.46	14.43	9.20
Nd	0.11	21.41	20.61	31.06	33.50	31.87	12.06	29.89	33.95	26.79	40.02	28.49	35.52	46.52	47.29	37.90	51.41	37.75	40.61	12.06	51.41	33.30
Sm	0.05	3.76	3.69	5.96	6.30	5.70	2.25	4.86	5.54	4.76	7.29	5.56	6.47	8.59	9.31	7.30	9.85	6.94	7.66	2.25	9.85	6.12
Eu	0.01	0.68	0.79	1.34	1.42	1.20	0.52	1.01	1.11	1.01	1.23	1.14	1.42	1.78	2.02	1.74	2.09	1.49	1.61	0.52	2.09	1.29
Gd	0.04	2.84	3.02	4.92	5.16	4.61	2.18	4.17	4.92	3.98	5.27	5.15	5.20	6.78	7.49	6.23	8.06	5.51	6.21	2.18	8.06	5.03
Tb	0.01	0.44	0.48	0.73	0.77	0.75	0.36	0.64	0.73	0.61	0.69	0.84	0.79	0.95	1.14	0.90	1.19	0.83	0.92	0.36	1.19	0.76
Dy	0.04	2.63	3.06	4.57	4.92	4.58	2.38	3.92	4.71	3.85	3.86	5.32	4.62	5.78	6.92	5.53	7.26	4.90	5.52	2.38	7.26	4.64
Ho	0.01	0.52	0.59	0.89	0.93	0.90	0.50	0.81	0.98	0.76	0.72	1.06	0.93	1.12	1.38	1.08	1.40	0.97	1.08	0.50	1.40	0.91
Er	0.04	1.62	1.77	2.52	2.74	2.81	1.52	2.43	2.95	2.29	2.09	3.04	2.70	3.37	4.18	3.31	4.13	2.82	3.21	1.52	4.18	2.72
Tm	0.01	0.24	0.25	0.38	0.40	0.41	0.23	0.34	0.41	0.33	0.31	0.43	0.39	0.49	0.59	0.48	0.59	0.40	0.46	0.23	0.59	0.39
Yb	0.01	1.75	1.78	2.68	2.81	2.84	1.54	2.18	2.74	2.29	2.11	2.69	2.65	3.21	3.93	3.18	3.81	2.65	3.03	1.54	3.93	2.64
Lu	0.01	0.28	0.26	0.40	0.41	0.42	0.24	0.33	0.41	0.34	0.33	0.41	0.41	0.50	0.60	0.49	0.55	0.39	0.46	0.24	0.60	0.40
REE		118.71	114.24	167.50	180.04	188.36	73.61	211.08	228.30	160.23	219.94	157.83	194.42	244.26	262.97	214.60	288.30	218.15	225.06	73.61	288.30	190.74
LREE		108.40	103.04	150.41	161.91	171.04	64.65	196.27	210.44	145.77	204.57	138.91	176.73	222.07	236.75	193.40	261.31	199.68	204.18	64.65	261.31	173.25
HREE		10.32	11.20	17.10	18.14	17.33	8.96	14.82	17.86	14.46	15.38	18.93	17.69	22.19	26.22	21.20	26.99	18.46	20.88	8.96	26.99	17.48
LREE/HREE		10.51	9.20	8.80	8.93	9.87	7.22	13.25	11.78	9.94	13.30	7.34	9.99	10.01	9.03	9.12	9.68	10.81	9.91	7.22	13.30	9.93
Ce/Ce*		0.94	0.80	0.95	1.06	0.82	0.90	1.17	1.11	0.97	0.92	0.97	0.96	1.06	1.12	1.09	0.96	0.95	1.00	0.80	1.17	0.94
Eu/Eu*		0.63	0.71	0.74	0.75	0.70	0.70	0.68	0.64	0.69	0.33	0.41	0.43	0.41	0.44	0.48	0.43	0.42	0.42	0.33	0.75	0.63
(La/Yb)_N_		10.98	11.25	9.28	8.70	12.24	7.78	15.73	13.50	11.18	17.11	8.33	11.06	10.32	8.77	9.16	11.54	13.28	11.20	7.78	17.11	10.98
(La/Sm)_N_		4.69	4.97	3.84	3.56	5.61	4.89	6.47	6.13	5.02	4.56	3.71	4.17	3.54	3.40	3.66	4.09	4.65	3.97	3.40	6.47	4.69
(Gd/Yb)_N_		1.31	1.38	1.48	1.49	1.31	1.14	1.55	1.45	1.39	2.02	1.55	1.59	1.71	1.54	1.59	1.71	1.69	1.67	1.14	2.02	1.31

d.l.: detection limits.

Ce/Ce*= (Ce_sample_/Ce_chondrite_)/(La_sample_/La_chondrite_)^1/2^(Pr_sample_/Pr_chondrite_)^1/2^.

Eu/Eu*= (Eu_sample_/Eu_chondrite_)/(Sm_sample_/Sm_chondrite_)^1/2^(Gd_sample_/Gd_chondrite_)^1/2^.

(La/Yb)_N_= (La_sample_/La_chondrite_)/(Yb_sample_/Yb_chondrite_).

(La/Sm)_N_= (La_sample_/La_chondrite_)/(Sm_sample_/Sm_chondrite_).

(Gd/Yb)_N_= (Gd_sample_/Gd_chondrite_)/(Yb_sample_/Yb_chondrite_).

#### 4.2.4 Correlations

The Pearson's correlation matrix provides insights into the behavior of chemical elements in the Sanaga valley alluvial clay deposits (Table 6). Correlations are deemed strong when the coefficient exceeds 0.70 in absolute value. Positive values indicate elements associated in similar processes, while negative coefficients suggest different enrichment processes. Apart from REE, SiO_2_ exhibits negative correlations with several elements (e.g., Al_2_O_3_, Fe_2_O_3_, LOI, Cr, V, Ni, Ga). Conversely, Al_2_O_3_ is strongly correlated with multiple elements (LOI, Cr, V, Ni, Sc, and Ga), indicative of potential associations in the clay fraction [2, 29]. Fe_2_O_3_ is linked to V and Co, while K_2_O is associated with Ba, Sr, and Rb. The notable correlation between TiO_2_ and Nb suggests the potential for rutile to contain a significant amount of Nb. Loss on ignition (LOI), which increases with the degree of weathering due to clay minerals formation, exhibits a strong affinity with V, Cr, Ni, and Ga. Among the trace elements suite: (1) Ba is correlated with Sr and Rb; (2) positive correlation is observed for Cr with V, Ni, Sc, and Ga; (3) V is linked to Ni, Ga, and Pb; (4) Cu is positively correlated with Sc and Ga; (5) positive correlations exist between Sr and Rb, Th and Hf, Sc and Ga. As anticipated, strong positive correlations are evident among rare earth elements. Additionally, Rb and Y are correlated with rare earth elements.

**Table 6:** Pearson's correlation matrix for major, trace and rare earth elements of the Nkoteng-Mbandjock alluvial clays.

	SiO_2_	Al_2_O_3_	Fe_2_O_3_	K_2_O	TiO_2_	LOI	Ba	Sr	Li	Rb	Th	Zr	Nb	U	Hf	Cr	V	Ni	Zn	Cu	Co	Sc	Y	Ga	Pb	REE	LREE	HREE
SiO_2_	1.00																											
Al_2_O_3_	**−0.93**	1.00																										
Fe_2_O_3_	**−0.85**	0.63	1.00																									
K_2_O	0.02	−0.28	0.20	1.00																								
TiO_2_	−0.13	0.16	−0.04	−0.45	1.00																							
LOI	**−0.93**	**0.97**	0.68	−0.33	0.26	1.00																						
Ba	−0.02	−0.25	0.25	**0.98**	−0.46	−0.29	1.00																					
Sr	0.06	−0.36	0.22	**0.96**	−0.40	−0.37	**0.97**	1.00																				
Li	−0.23	0.49	−0.20	−0.35	0.15	0.38	−0.40	−0.55	1.00																			
Rb	0.40	−0.59	−0.18	**0.70**	−0.22	−0.57	**0.71**	**0.70**	−0.33	1.00																		
Th	−0.11	0.12	−0.03	0.06	0.31	0.13	−0.03	−0.06	0.36	0.22	1.00																	
Zr	0.47	−0.68	−0.17	0.63	−0.10	−0.63	0.54	0.65	−0.58	0.67	0.13	1.00																
Nb	−0.06	0.13	−0.12	−0.53	**0.97**	0.22	−0.54	−0.48	0.21	−0.22	0.23	−0.15	1.00															
U	−0.31	0.16	0.22	0.48	0.37	0.17	0.49	0.45	0.04	0.54	0.49	0.22	0.28	1.00														
Hf	0.43	−0.66	−0.11	0.68	−0.13	−0.62	0.59	0.69	−0.59	0.67	0.18	**0.98**	−0.19	0.26	1.00													
Cr	**−0.79**	**0.81**	0.54	−0.36	0.52	**0.86**	−0.35	−0.39	0.37	−0.50	0.12	−0.45	0.52	0.26	−0.47	1.00												
V	**−0.91**	**0.86**	**0.75**	−0.12	0.38	**0.88**	−0.07	−0.15	0.29	−0.34	0.16	−0.43	0.33	0.43	−0.42	**0.83**	1.00											
Ni	**−0.84**	**0.86**	0.60	−0.17	0.03	**0.87**	−0.16	−0.23	0.30	−0.46	0.03	−0.44	0.03	0.11	−0.46	**0.82**	**0.72**	1.00										
Zn	−0.46	0.39	0.31	0.48	−0.03	0.35	0.40	0.29	0.45	0.28	0.49	0.15	−0.04	0.58	0.17	0.36	0.46	0.43	1.00									
Cu	−0.68	0.65	0.51	0.05	0.03	0.69	0.02	−0.04	0.26	−0.07	0.30	−0.05	0.02	0.36	−0.10	0.68	0.66	**0.84**	0.68	1.00								
Co	−0.68	0.47	**0.73**	0.42	−0.07	0.49	0.44	0.42	−0.15	0.02	−0.01	0.10	−0.10	0.35	0.12	0.55	0.55	0.69	0.57	0.64	1.00							
Sc	−0.60	**0.70**	0.28	−0.38	0.35	0.76	−0.36	−0.42	0.37	−0.29	0.24	−0.37	0.37	0.25	−0.45	**0.79**	0.65	**0.80**	0.35	**0.81**	0.35	1.00						
Y	0.26	−0.29	−0.34	0.30	0.06	−0.23	0.23	0.28	0.00	0.56	0.42	0.52	0.09	0.43	0.43	−0.04	−0.22	−0.04	0.36	0.33	0.04	0.31	1.00					
Ga	**−0.90**	**0.98**	0.57	−0.28	0.22	**0.96**	−0.26	−0.38	0.56	−0.51	0.20	−0.63	0.21	0.24	−0.62	**0.85**	**0.88**	**0.87**	0.49	**0.75**	0.46	**0.79**	−0.14	1.00				
Pb	−0.64	0.57	0.43	−0.04	0.55	0.63	0.03	0.00	0.17	−0.05	0.16	−0.35	0.53	0.68	−0.35	0.67	**0.73**	0.43	0.30	0.39	0.35	0.58	0.14	0.62	1.00			
REE	0.57	−0.61	−0.50	0.20	0.11	−0.51	0.15	0.22	−0.22	**0.70**	0.38	0.62	0.14	0.32	0.55	−0.32	−0.49	−0.39	0.04	−0.01	−0.25	0.02	**0.85**	−0.48	−0.06	1.00		
LREE	0.59	−0.62	−0.51	0.17	0.11	−0.53	0.12	0.20	−0.23	0.69	0.37	0.62	0.14	0.29	0.55	−0.34	−0.51	−0.41	0.01	−0.03	−0.27	0.00	**0.83**	−0.50	−0.08	**1.00**	1.00	
HREE	0.27	−0.35	−0.30	0.42	0.10	−0.29	0.37	0.41	−0.08	**0.70**	0.47	0.55	0.09	0.59	0.49	−0.10	−0.22	−0.14	0.36	0.22	0.06	0.18	**0.96**	−0.21	0.22	**0.88**	**0.86**	1.00

## 5 Discussion

### 5.1 Source weathering conditions

Strong relationships exist between weathering intensities and the chemical dynamics of the upper crust. Throughout the weathering process, highly mobile elements such as Na and K tend to exit sediments, while the least mobile elements, such as Fe and Al, remain in the residual sediments. High loss on ignition (LOI) value is generally stemmed from abundant volatile losses during heating. The moderate Al_2_O_3_ contents, coupled with high LOI and Chemical Index of Alteration (CIA) values in the Sanaga valley alluvial clay deposits, can be attributed to the prevalence of clay minerals [21], indicative of a high degree of weathering.

**Fig. 6:**
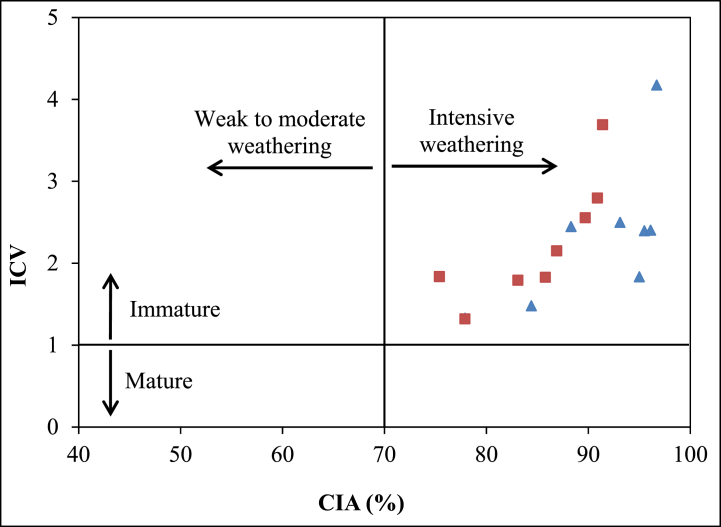
Geochemical diagrams of Index of Compositional Variability (ICV) vs Chemical Index of Alteration (CIA) of the Nkoteng-Mbandjock alluvial clays [50].

In a broader context, fresh rocks typically exhibit alteration indices (CIA and PIA) values of about 50%, potentially escalating to 100% after undergoing weathering. Low values indicate inactive chemical reactions, often associated with weak weathering under cold or arid conditions [24]. Conversely, high values reflect active chemical reactions linked to robust weathering conditions in warmer environments. The calculated CIA values in the Sanaga valley alluvial clay deposits showcase a considerable range (76–97%), with a mean value of 88% (Table 3). Sediments originating from the Mbandjock site (average 91%) exhibit a higher degree of weathering compared to those from Nkoteng (average 88%). It is noteworthy that while both CIA and PIA values demonstrate a similar trend, the indices collectively indicate a high degree of weathering, corroborated by the plot of Index of Compositional Variability (ICV) *vs.* CIA (Fig. 6).

### 5.2 Classification and maturity

**Fig. 7:**
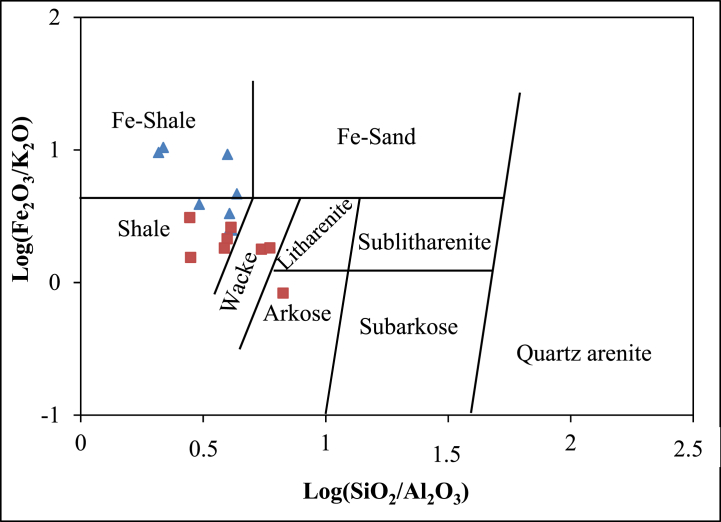
Geochemical classification diagram of the Nkoteng-Mbandjock alluvial clays using log ratios of SiO_2_/Al_2_O_3_–Fe_2_O_3_/K_2_O [11]. Triangles represent samples from Mbandjock and squares represent samples from Nkoteng.

The Herron diagram, utilizing the logarithmic ratios of four major elements (Fe, Al, Si, and K) as proposed by Herron [11], emerges as a reliable tool for sediment classification. The logarithmic ratios, specifically Log SiO_2_/Al_2_O_3_ *vs.* Log Fe_2_O_3_/K_2_O, provide a comprehensive framework. In this diagram, the majority of samples from the Sanaga valley alluvial clay deposits fall in the field of shale and Fe-shale composition, as illustrated in Fig. 7. Notably, a single sample from Nkoteng diverges into the arkose field. The low Na_2_O/K_2_O values observed in the Sanaga valley alluvial clay deposits are indicative of immature sediments. This immaturity is further confirmed by the Index of Compositional Variability (ICV) and their SiO_2_/Al_2_O_3_ ratios, both falling below 5 [30]. Such samples are likely sourced from stable settings [2]. According to Cullers and Podkovyrov [31], an ICV>1 indicates an immature composition with no recycled inputs. The calculated Chemical Index of Alteration (CIA), Plagioclase Index of Alteration (PIA), and mineralogical composition collectively suggest intense weathering of source materials. This observed behavior aligns with the humid and warm tropical climate prevailing in the Sanaga basin [32].

**Fig. 8:**
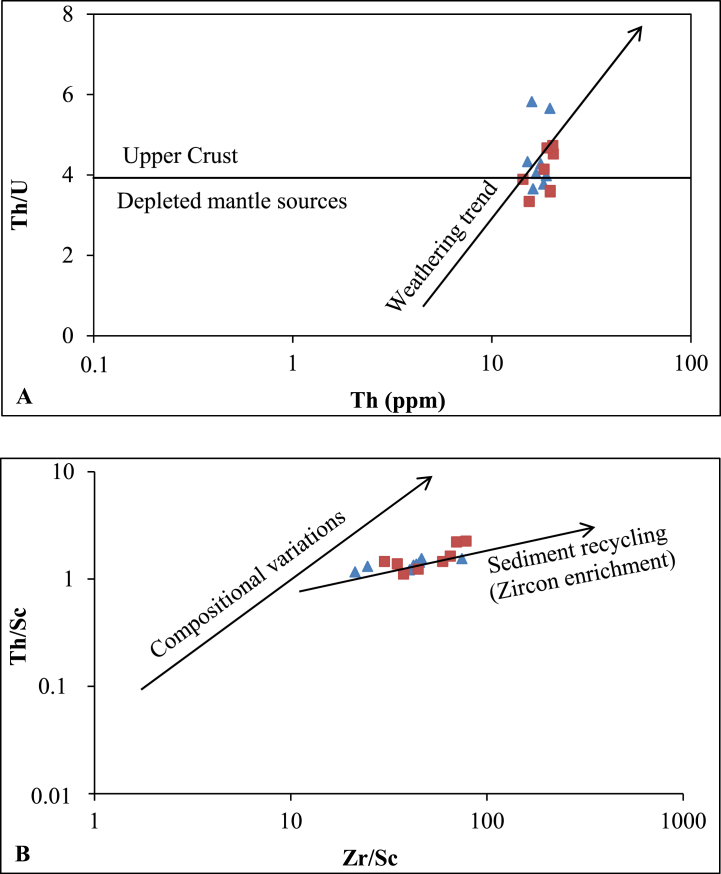
Discrimination bivariate plots illustrating weathering and sediment recycling: A) Th/U vs Th plot; B) Th/Sc vs Zr/Sc plot [33] of the Nkoteng-Mbandjock alluvial clays. Triangles represent samples from Mbandjock and squares represent samples from Nkoteng.

The Th/U values portray a contrasting composition and further indicate the complete weathering of source rocks (see Fig. 8A). Positively correlated between Zr/Sc and Th/Sc ratios, along with the gradual increase in Zr/Sc values, suggest a scenario of sediment recycling (refer to Fig. 8B). Indeed, sediment reworking typically leads to a rapid increase in Zr/Sc ratios [33].

### 5.3 Depositional environment

Trace element ratios, such as V/Cr, U/Th, and the authigenic U index (AU = XU-(YTh)/3; Table 4), serve as valuable tools for discussing the paleoenvironment prevailing during deposition. Specifically, U/Th ratios greater than 1.25 and V/Cr ratios exceeding 4.5 are indicative of an anoxic environment, while U/Th ratios below 0.75 and V/Cr ratios less than 2 suggest oxic conditions [34,35]. The deposition of the Sanaga valley alluvial clay deposits is established to have occurred under oxic conditions, with an average U/Th ratio of 0.24 and V/Cr ratio of 1.17 (Table 3). This oxic depositional environment is further corroborated by the low authigenic U values, averaging −1.67 (Table 3). According to Jones and Manning [34], low authigenic U values (<5) signify an oxic depositional condition, whereas higher values (>5) suggest anoxic conditions.

Despite the generally consistent geochemical behavior of Rare Earth Elements (REE) and their low solubility in natural conditions, Ce is influenced by variations in redox potential (Eh) and can serve as an indicator of oxidizing conditions during deposition. Cerium exists in the Ce^3+^ form, similar to other REE, and as Ce^4+^ in oxidizing environments in the supergene environment [36]. The Ce^4+^ state remains more stable, forming cerianite (CeO_2_) or on the surfaces of Fe- and Mg-oxihydroxides, generating positive Ce-anomalies. The Sanaga valley alluvial clay deposits show both negative and positive Ce-anomaly. This indicates oxidizing and reducing depositional conditions in the Sanaga valley alluvial clay deposits. This divergent behavior of Ce may be attributed to post-depositional solutions and solids, which can generate negative Ce-anomalies at specific levels in the deposit [37]. The regular flooding observed in the lowlands of the Sanaga basin could be potentially responsible for these post-depositional changes. This can also be explained by a variation of depositional environment in time as revealed by the vertical color variation of the different layers of the deposits.

### 5.4 Provenance

**Fig. 9:**
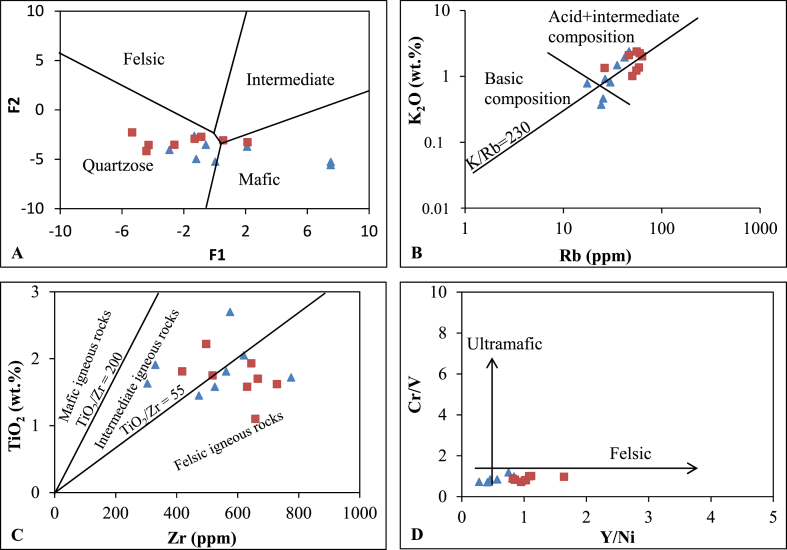
Bivariate plots for provenance of the Nkoteng-Mbandjock alluvial clays: (A) Major element provenance discriminant plot [41]; (B): K_2_O vs Rb [42] with K/Rb ratio = 230 (main trend of Shaw [43]); (C) Zr vsTiO_2_ [51]; (D) Cr/V vs Y/Ni plot [13, 33].

Geochemical elements play a pivotal role in identifying sedimentary provenances as they faithfully reflect the compositions of source rocks [38,39]. Aluminum (Al) and titanium (Ti) oxides, being less fractionated during surface processes such as weathering, transport, and diagenesis, provide valuable insight into sediment provenances through Al_2_O_3_/TiO_2_ ratios [40]. These ratios increase progressively from mafic (Al_2_O_3_/TiO_2_ = 3–8; SiO_2_ = 45–52 wt%) to intermediate (Al_2_O_3_/TiO_2_ = 8–21; SiO_2_ = 53–66 wt%) and felsic (Al_2_O_3_/TiO_2_ = 21–70; SiO_2_ = 66–76 wt%) rocks [40]. In this study, Al_2_O_3_/TiO_2_ values range from 6 to 15 (average 10), accompanied by elevated SiO_2_ contents (51–77 wt% with an average of 65 wt%), indicative of felsic and intermediate sources. The discriminant plot, based on elemental contents, reveals that the samples from the Sanaga valley alluvial clay deposits fall into two mixed provenances-mafic and especially quartzose recycled areas (Fig. 9A). The K_2_O–Rb plot places the samples predominantly in the acid and intermediate source fields near the trend of differentiated magmatic suites (K_2_O/Rb = 230) (Fig. 9B). The Zr *vs.* TiO_2_ diagram also positions the deposits in two mixed provenances-intermediate and felsic igneous rocks (Fig. 9C). The influence of mafic and intermediate rocks may be attributed to the presence of small mafic rocks in the Sanaga basin, such as amphibolites and micaschists.

The transportation, pre- and post-depositional weathering processes can alter the major element contents of the source rocks [44]. Sediments resulting from high post-depositional chemical weathering tend to concentrate residual stable minerals during surface processes [45,55]. Immobile trace elements associated with mafic (Co, Cr, Sc, and Ni) and felsic (La, Y, Th, Zr, and REE) sediment compositions prove to be effective tools in discerning the nature of the source rock [[45], [46], [47], [48]]. For fine sediments in this study, the contents in Th and Zr are high (synonym of zircon heavy mineral) just like the concentrations in Sc [55]. Elemental ratios such as Th/Sc, Ni/Co, and Zr/Sc exhibit high values (>1), while Cr/V, Y/Ni, and U/Th ratios display low values (<1). These ratios collectively suggest that the Sanaga valley alluvial clay deposits originate from felsic sources, a conclusion supported by the high LREE/HREE ratios and negative europium anomaly [38,55].

**Fig. 10:**
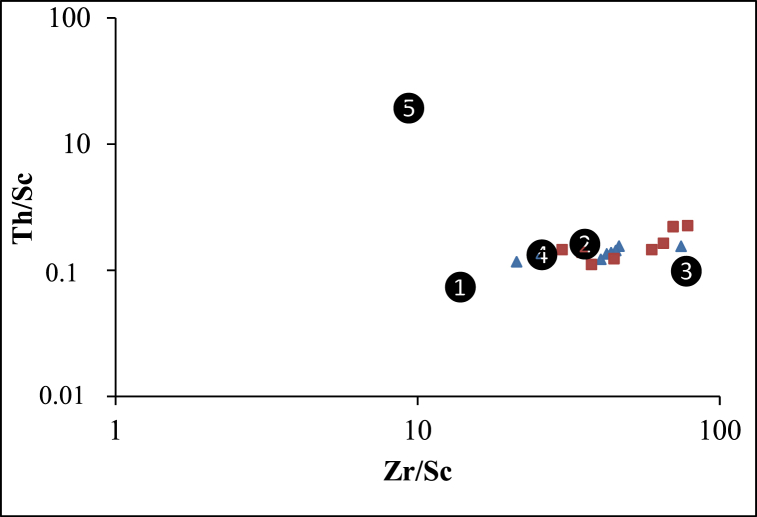
Th/Sc–Zr/Sc plot for geochemical characterization of rock sources with (1) average upper continental crust (UCC, [52]), (2) micaschist, (3) gneiss, (4) granite [53] and (5) mylonite [54].

Furthermore, the Cr/Y *vs.* Y/Ni plot confirms the felsic sources, as indicated by their relatively low Cr/V values and high Y/Ni ratios (Fig. 9D). This aligns with the geological setting, which predominantly features felsic rocks in the Sanaga basin (Fig. 1). The regional geology serves as a crucial and reliable tool for discriminating source rocks in provenance studies [20]. Comparisons of sediment compositions with those of regional rocks place the data close to the compositions of gneiss, granite, and micaschist in the Th/Sc *vs.* Zr/Sc distribution diagram (Fig. 10), affirming the felsic source of the sediments with a contribution from mafic rocks, as indicated by other discriminant plots (Fig. 9).

## 6 Conclusions

Based on the comprehensive analysis of mineralogical and geochemical data from the Nkoteng-Mbandjock alluvial clays in the Sanaga valley deposits (Central Africa), the following key conclusions are drawn:

a. The clayey alluvial sediments in the Nkoteng-Mbandjock areas predominantly consist of quartz, kaolinite, gibbsite, goethite/hematite, illite, and rutile;
b. These sediments are the result of extensive weathering of felsic and mafic source rocks, exhibiting a composition akin to shale, Fe-shale, and wacke;
c. The sediments are classified as immature, and suggest a depositional environment of recycled inputs in stable settings under oxic conditions;
d. Post-depositional activities have led to modifications in the oxidizing conditions at certain levels in the deposit, reflecting dynamic environmental influences over time.

These conclusions provide valuable insights into the geological history and environmental conditions that shaped the Nkoteng-Mbandjock alluvial clays in the Sanaga valley, contributing to our understanding of sediment weathering, provenance and depositional processes in this region of Central Africa.

## Data availability statement

Data obtained during the study are included in the manuscript.

## CRediT authorship contribution statement

**Elisé Sababa:** Writing – review & editing, Writing – original draft, Methodology, Data curation, Conceptualization. **Natanael Tehna:** Writing – original draft, Formal analysis. **Beyanu Anehumbu Aye:** Writing – review & editing, Writing – original draft, Funding acquisition. **Morine-Majolie Manfotang Chiozem:** Writing – original draft, Data curation. **Armel Zacharie Ekoa Bessa:** Writing – review & editing, Writing – original draft, Methodology, Data curation, Conceptualization. **Ehbeudeu Kanewene:** Writing – original draft, Methodology. **Njilah Isaac Konfor:** Writing – review & editing, Supervision, Project administration, Conceptualization.

## Declaration of competing interest

The authors declare that they have no known competing financial interests or personal relationships that could have appeared to influence the work reported in this paper.
